# Clinical specificities of depression with overvalued ideas in slow progressive schizophrenia

**DOI:** 10.1192/j.eurpsy.2022.1998

**Published:** 2022-09-01

**Authors:** A. Barkhatova, O. Kolyago

**Affiliations:** Mental Health Research Center, Department Of Endogenous Mental Disorders And Affective Conditions, Moscow, Russian Federation

**Keywords:** Overvalued ideas, depression, schizophrenia

## Abstract

**Introduction:**

Despite the high interest in affective aspects of endogenous depression, the question of overvalued ideas remains unclear.

**Objectives:**

To determine the clinical features of depression with overvalued ideas in the context of slow progressing schizophrenia.

**Methods:**

The study involved 102 people, including 62 women and 40 men diagnosed with slow progressing schizophrenia, with moderate or severe depression, with the predominance of overvalued ideas (F21 in ICD-10). The control group had 110 people with similar distrubution by sex and age all diagnosed with a depressive state formed in the context of slow progressing schizophrenia, without overvalued ideas. Research methods were mainly clinical-psychopathological and clinical-catamnestic.

**Results:**

In the majority of observations (62.5%) in the comparison group, showed a uncomplicated form of depression, only limited to affective level. In the (37.5%) left, depression was characterized by a structural polymorphism, in addition to affective disorders, there were other psychopathological syndromes observed. In the main group, an inverse relationship was observed: uncomplicated depression in 29.2% of cases, while the overwhelming majority of cases (70.8%) experienced atypical depressions, disharmony of the affective triad, as well as an increase in the concomitance of neurotic and psychopathic symptoms were observed.

**Conclusions:**

The cohort of patients with slow progressive schizophrenia with depression is heterogeneous, while the features of the course of the disease correlate both with the presence or absence of overvaluated formations in the picture of depression, and with the features of the modification of the overvaluated ideas during the disease.

**Disclosure:**

No significant relationships.

